# The IRE1α–XBP1s Arm of the Unfolded Protein Response Activates N-Glycosylation to Remodel the Subepithelial Basement Membrane in Paramyxovirus Infection

**DOI:** 10.3390/ijms23169000

**Published:** 2022-08-12

**Authors:** Yingxin Zhao, Dianhua Qiao, Melissa Skibba, Allan R. Brasier

**Affiliations:** 1 Department of Internal Medicine, University of Texas Medical Branch, Galveston, TX 77555-1060, USA; 2 Department of Medicine, School of Medicine and Public Health (SMPH), University of Wisconsin-Madison, Madison, WI 53705, USA; 3 Institute for Clinical and Translational Research (ICTR), University of Wisconsin-Madison, Madison, WI 53705, USA

**Keywords:** unfolded protein response, IRE1α, XBP1, hexosamine biosynthetic pathway, N-glycosylation, extracellular matrix

## Abstract

Respiratory syncytial virus (RSV) causes severe lower respiratory tract infections (LRTI) associated with decreased pulmonary function, asthma, and allergy. Recently, we demonstrated that RSV induces the hexosamine biosynthetic pathway via the unfolded protein response (UPR), which is a pathway controlling protein glycosylation and secretion of the extracellular matrix (ECM). Because the presence of matrix metalloproteinases and matricellular growth factors (TGF) is associated with severe LRTI, we studied the effect of RSV on ECM remodeling and found that RSV enhances the deposition of fibronectin-rich ECM by small airway epithelial cells in a manner highly dependent on the inositol requiring kinase (IRE1α)–XBP1 arm of the UPR. To understand this effect comprehensively, we applied pharmacoproteomics to understand the effect of the UPR on N-glycosylation and ECM secretion in RSV infection. We observe that RSV induces N-glycosylation and the secretion of proteins related to ECM organization, secretion, or proteins integral to plasma membranes, such as integrins, laminins, collagens, and ECM-modifying enzymes, in an IRE1α–XBP1 dependent manner. Using a murine paramyxovirus model that activates the UPR in vivo, we validate the IRE1α–XBP1-dependent secretion of ECM to alveolar space. This study extends understanding of the IRE1α–XBP1 pathway in regulating N-glycosylation coupled to structural remodeling of the epithelial basement membrane in RSV infection.

## 1. Introduction

Respiratory syncytial virus (RSV), a human-adapted enveloped negative-sense orthopneumovirus, is responsible for seasonal outbreaks of respiratory tract infections worldwide [1]. Infecting more than 37 million people annually, RSV is the most common cause of pediatric hospitalization [2] and is responsible for 1/3 of lower respiratory tract infections (LRTIs) globally [3]. A major target responsible for LRTI pathogenesis is the lower airway epithelial cell, which is a cell type that produces a robust innate antiviral response consisting of secretion of cytokine [4,5], interferon [6], and damage-associated patterns [7], resulting in epithelial giant cell formation and necrosis, mucous plugging, ventilation–perfusion mismatching, and acute hypoxic respiratory failure [8].

Prospective studies of children with severe LRTIs have shown that these infections are associated with decreased pulmonary function, asthma, and allergy over long-term follow-up [9,10,11]. The mechanisms for these long-term effects are currently unclear; however, remodeling of the basal lamina may play a role, based on several lines of evidence: (i) Children with severe LRTI express more significant amounts of ECM remodeling proteins, including matrix metalloproteinases (MMPs) in their nasal secretions [12]; (ii) MMP9 activity is increased in children with RSV LRTI requiring mechanical ventilation [13]; (iii) RSV infections in neonatal mice are associated with enhanced hyaluronan deposition [14]; and (iv) RSV is a potent inducer of TGFβ secretion and MMP9 expression in lower airway epithelial cells driving profibrotic myofibroblast transition [15,16]. However, the molecular details of how RSV restructures the ECM are not fully understood. 

We recently reported a new mechanism that links viral-induced unfolded protein response (UPR) with glucose metabolic reprogramming [16,17,18]. Here, RSV infection activates the inositol-requiring protein 1 (IRE1α)–X-box-binding protein 1 (XBP1) axis of UPR coupled to expression of rate-limiting enzymes in the hexosamine biosynthesis pathway (HBP), shifting the glucose flux from glycolysis to uridine diphosphate N-acetylglucosamine (UDP-GlcNAc) production. Furthermore, our mechanistic studies showed that RSV enhances XBP1 binding to the super-enhancer of the HBP rate-limit enzyme glutamine-fructose-6-phosphate aminotransferase 2 (*GFPT2*), promoting RNA Polymerase II engagement to the *GFPT2* gene [17]. In vivo, the murine respiratory virus Sendai virus (SeV) also induces the activation of HBP in mouse lungs in an IRE1α-dependent manner. Collectively, these studies indicate that the IRE1α–XBP1 arm of UPR mediates paramyxovirus-induced cellular glucose metabolic reprogramming [17]. UDP-GlcNAc is the final product of HBP and is the essential substrate for protein N-glycosylation. However, the effects of enhanced protein N-glycosylation in viral infection and ECM production are not fully understood.

To advance the field, we explored the effects of RSV infection on metabolic reprogramming and airway remodeling in this study. We found that RSV increased the production of a fibronectin-rich basal lamina dependent on the IRE1α–XBP1 pathway. To understand this process mechanistically, we applied pharmacoproteomics of protein N-glycosylation and secretion. RSV induces the secretion of N-linked ECM modifying proteins, including MMPs, lysyl oxidase, and major components of the basal lamina. The in vitro finding was validated by proteomics analysis of bronchoalveolar lavage fluid (BALF) of mice infected with murine respiratory virus, where glycoprotein secretion of ECM components and innate and adaptive immune proteins were produced in an IRE1α-dependent manner. These data indicate that the paramyxovirus-induced IRE1α–XBP1 arm of UPR is central to protein N-glycoprotein and the secretion of ECM proteins and ECM-modifying enzymes, providing unique insights into structural remodeling induced by viral airway infections. 

## 2. Results

### 2.1. RSV Infection Remodels the Epithelial Basement Membrane

Our previous studies found that RSV infection induces rapid activation of the IRE1α–XBP1 arm of UPR in primary small airway epithelial cells [16,17]. The formation of spliced XBP1 (XBP1s) is required not only for activation of the HBP but also for the expression of mesenchymal transition (EMT) through the Snail family transcriptional repressor 1 (SNAI1) [17].

**Figure 1:**
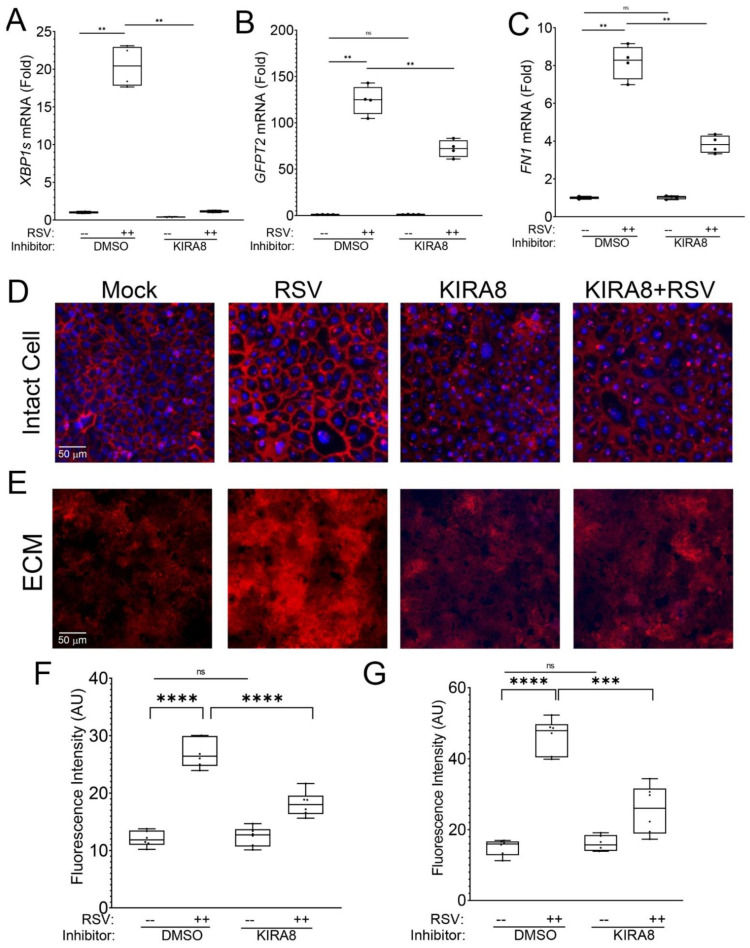
RSV induces ECM remodeling via the IRE1α–XBP1 arm of UPR. hSAECs were treated with solvent control (DMSO) or KIRA8 (10 µM) and mock- or RSV infected (MOI = 1, 24 h). Total RNA was extracted and analyzed by Q-RT-PCR for (**A**) XBP1 splicing; (**B**) *GFPT2*; and (**C**) *FN1.* For each graph, fold change mRNA relative to solvent-treated mock-infected cells is shown. ***, *p* < 0.001; n.s., not significant. (**D**), hSAECs were cultured on PDL-gelatin coated coverslips until confluent, which was followed by treatment with solvent or KIRA8. Cells were then mock or RSV-infected (MOI = 1, 24 h). The cells were fixed and stained for extracellular FN1 without permeabilization. Nuclei were then stained with DAPI. Red, FN1. Blue, DAPI. Scale bar 50 µm shown. (**E**). Identically treated plates were decellularized and stained for FN1 and imaged. (**F**,**G**), Quantitation of the FN1 fluorescence intensity by FIJI. The data points and mean from three independent experiments are presented. **, *p* < 0.01; ***, *p* < 0.001; ****, *p* < 0.0001; n.s., not significant.

KIRA8 is a potent small-molecule inhibitor of IRE1α that selectively reduces XPB1s formation without affecting the other signaling arms of the UPR, ATF6, or CHOP [17,19]. In this study, we confirmed that the IRE1α–XBP1 signaling pathway was required for *GFPT2* and fibronectin (*FN1*) expression. Human small airway epithelial cells (hSAECs) were mock- or RSV-infected in the presence or absence of KIRA8 and RNA analyzed by Q-RT-PCR. We confirmed that RSV was a potent inducer of XBP1 splicing in solvent-only treated cells, where a 20-fold increase in *XBP1s* formation was observed (*p* < 0.001, Figure 1A). Importantly, this RSV induction was reversed to that of solvent-treated mock-infected cells by KIRA8 treatment (Figure 1A). We also observed a 120-fold increase in *GFPT2* expression in solvent-treated cells relative to mock-infected cells that was reduced to 72-fold by KIRA8 treatment (*p* < 0.01, Figure 1B). Importantly, there was no significant difference between solvent-treated, mock infected cells and KIRA8-treated, mock-infected cells (Figure 1B). Similarly, in solvent-treated cells, RSV infection produced an 8.2-fold induction of *FN1*, which was an induction that was reduced to 3.2-fold by KIRA8 treatment (Figure 1C). These data confirmed that the robust activation of the IRE1α–XBP1 pathway by RSV was inhibited by KIRA8.

To further understand the role of the induced UPR on cell-associated FN1, hSAECs cultured on poly-D-lysine (PDL)-gelatin-coated slides were infected with sucrose cushion-purified RSV in the absence or presence of KIRA8. In this experiment, fixed cells were stained with anti-fibronectin (FN1) Ab in the absence of permeabilization and imaged by microscopy. We observed that the differentiated airway epithelial cells form a rich intercellular network of FN1 (Figure 1D). Interestingly, upon RSV infection, the abundance of the FN1 in the intercellular meshwork was significantly enhanced ≈2.2-fold (Figure 1D; quantitation in Figure 1F). KIRA8 treatment alone had no discernible effect on FN1 distribution relative to solvent-treated mock-infected cells (Figure 1D,F). By contrast, in RSV-infected cells treated with KIRA8, the abundance of FN1 was reduced nearly to that of control (Figure 1D,F).

To examine the role of IRE1α–XBP1s on secreted ECM, identically treated hSAECs were selectively removed to examine the ECM, and the native basal lamina was fixed and stained with anti-FN1 Ab. We observed that RSV infection enhanced FN1 deposition into the ECM (Figure 1E,G). In a manner similar to our observations on the RSV induction of cell-associated FN1, we found that FN1 deposition into the ECM was also blocked by KIRA8 (Figure 1D–F).

After finding that in uninfected cells, KIRA8 has no effect on GFPT2 and FN1 expression as well as detectable effects on FN1 distribution or ECM deposition, we conclude that the IRE1α pathway is active not in the basal state but primarily in response to RSV infection. For these reasons, in subsequent studies, we focus on the effects of KIRA8 in response to RSV infection.

FN1 is a ‘master regulator’ of ECM assembly by polymerizing other ECM components, including collagen [20]. From these data, we concluded that RSV infection induced the production and secretion of FN1-containing ECM. To comprehensively understand how RSV restructures the epithelial component of the basal lamina and how the IRE1α–XBP1 arm of UPR regulates this process, the proteome, secretome, and N-glycosylated proteins were quantified by MS using a label-free approach.

### 2.2. Proteomics Analysis of the Effect of the IRE1α–XBP1 Arm of UPR on RSV-Induced Host Response

**Figure 2:**
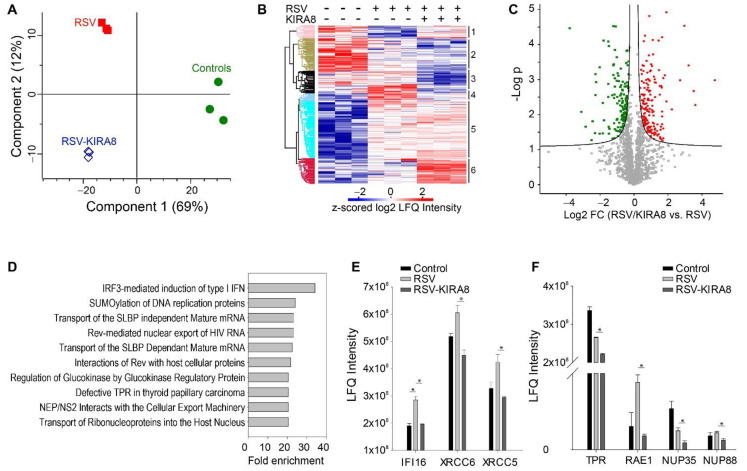
Proteomics analysis of hSAECs infected with RSV in the presence or absence of KIRA8. hSAECs were infected with RSV at 1.0 MOI for 24 h in the presence or absence of KIRA8 (10 µM). The proteins were analyzed with label-free LC-MS/MS. (**A**) Principal component analysis of significant proteins (ANOVA with permutation-based FDR < 0.01). Green circle, controls; red square, RSV infection; blue diamond, RSV infection + KIRA8 treatment. (**B**) Unsupervised hierarchical cluster analysis of 813 significant proteins. The colors of the heatmap represent the z-scored normalized log2 LFQ intensity of each protein. (**C**) Volcano plot of proteins (RSV+KIRA8 vs. RSV). Significantly proteins. Red circle, proteins upregulated by KIRA8; green square, proteins downregulated by KIRA8. (**D**) Top Panther Reactome pathways activated by RSV infection but blocked by KIRA8 (FDR < 0.05%). (**E**) Protein expression of IRF3-mediated type I IFN genes. (**F**) Expression of proteins involved in the interaction of viral structure protein NS2 with the cellular export machinery. Student’s *t*-test with permutation correction, *, q < 0.05.

To understand the role of the IRE1α–XBP1 pathway in the host response, we first analyzed the global changes in the proteome of hSAECs infected with RSV in the presence or absence of KIRA8 with untreated cells as the control. This analysis of hSAEC proteome quantified 1530 proteins (Supplemental Table S1). Among them, the abundance of 813 proteins showed a group-wise difference (multiple-sample ANOVA test with permutation-based FDR correction, q-value < 0.05 was statistically significant). Then, to assess the reproducibility of protein quantification among the replicates and obtain an overview of the proteome profiles obtained from the three experimental conditions, we performed principal component analysis (PCA) using 813 significant proteins. As shown in Figure 2A, each group’s replicates are clustered together, indicating that the LC-MS quantification of proteins is highly reproducible. Additionally, the PCA scatter plot of protein abundance has three completely separated clusters representing three experimental conditions (Figure 2A), suggesting that RSV infection and inhibition of IRE1α have distinct effects on protein expression changes.

The unsupervised hierarchical cluster analysis of 813 significant proteins resulted in six significant clusters (Figure 2B). GO annotation enrichment analysis for proteins in each cluster identified a total of 94 terms (Benj. Hoch. FDR < 0.02) (Supplemental Table S2). Cluster 4 mostly segregates proteins induced by RSV and blocked by the IRE1α inhibitor. GO annotation enrichment analysis of these proteins reveals that endoplasmic reticulum (ER)-resident lumen proteins were enriched in this cluster (enrichment fold 5.17, *p*-value = 0.000173, Benj. Hoch. FDR 0.019). ER stress markers, such as heat shock proteins (HSP)-A5/Bip, -90B1, and PDIA3, were induced by RSV infection and restored to the untreated level by KIRA8. Of these, HSPA5/Bip is an ER luminal protein that plays a key regulatory role in initiating the IRE1α–XBP1s pathway. This finding extends our previous report that HSPA5/Bip is activated at the gene expression level by RSV infection [17].

To investigate the effect of inhibiting the IRE1α–XBP1 arm of the UPR on the RSV-induced host response, we compared the protein expression in RSV-infected hSAECs in the presence or absence of KIRA8 and identified 169 upregulated proteins and 140 down-regulated (Student’s *t*-test with permutation-based FDR 5%) (Figure 2C, Supplemental Table S1). Pathway analysis of 169 upregulated proteins identified that 166 pathways were affected by KIRA8 (Fisher Exact FDR 5%) (Supplemental Table S3). The top 3 most enriched pathways are type I hemidesmosome assembly, the formation of tubulin folding intermediates by CCT/TriC, and the uptake and function of anthrax toxins. Panther Reactome pathway analysis of 140 proteins downregulated by KIRA8 shows that 67 Reactome pathways were affected (Fisher Exact FDR 5%). The pathways related to viral replication and host response were highly enriched, including IRF3-mediated induction of type I IFN, the interaction of viral structure protein NS2 with the cellular export machinery, viral structure protein NS1-mediated host pathways, and viral mRNA synthesis (Figure 2D). We found that RSV induced the expression of IRF3-mediated type I IFN genes, such as interferon alpha-inducible protein 6 (IFI6), X-ray repair cross-complementing protein 5 (XRCC5/Ku86), and X-ray repair cross-complementing protein 5 (XRCC6/Ku70), and this induction was blocked by KIRA8 (Figure 2E). In addition, we found that the expression of several proteins involved in the nuclear export pathway was regulated by KIRA8, including nucleoprotein TPR, mRNA export factor (RAE1), nucleoporin NUP35, and NUP88. Here, we found that KIRA8 treatment significantly reduced the expression of these proteins in RSV-infected cells (Figure 2F). Previous reports suggest that the interaction between virus nonstructural proteins NS1 and NS2 and the nuclear export pathway is essential for the nuclear export of virus ribonucleoprotein (RNP) complexes and virus generation [21], suggesting that the IRE1α–XBP1 arm of UPR may play a role in regulating the interaction of viral proteins with host proteins and innate immune response.

### 2.3. IRE1α–XBP1 Arm of UPR Regulates N-Glycosylation in RSV-Induced hSAECs 

**Figure 3:**
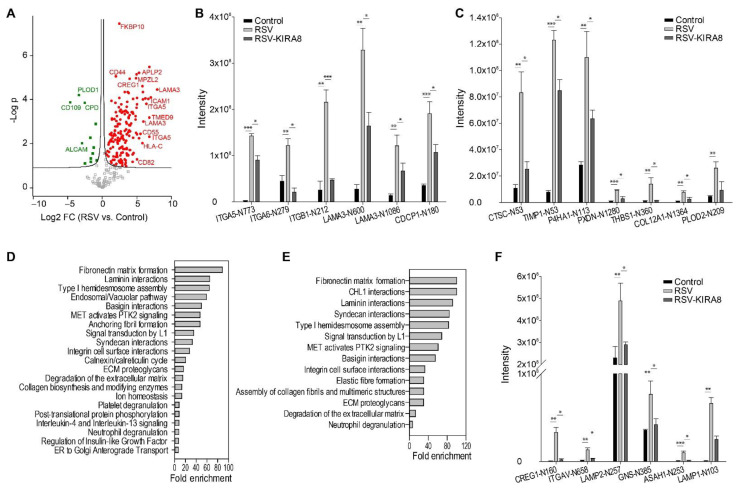
Proteomics analysis of N-glycosylation in hSAECs infected with RSV in the presence or absence of KIRA8. hSAECs were infected with RSV at 1.0 MOI for 24 h in the presence or absence of KIRA8 (10 µM). The N-glycosylated peptides were enriched with lectins and then analyzed with label-free LC-MS/MS. (**A**) Volcano plot of N-glycosylated peptides (RSV vs. Control). Red circle, N-glycoproteins upregulated by RSV; green square, N-glycoproteins downregulated by RSV infection. (**B**,**C**) Some N-glycosylated peptides strongly induced by RSV infection and regulated by the IRE1α–XBP1 arm of UPR are shown (Student’s *t*-test with permutation FDR < 0.05). (**D**) Panther Reactome pathways activated by RSV infection (FDR < 0.05%). (**E**) Panther Reactome pathways activated by RSV infection and attenuated by KIRA8 (FDR < 0.05%). (**F**) N-glycosylation of proteins involved neutrophil degranulation, which was regulated by the IRE1α–XBP1 arm of UPR. Student’s *t*-test with Permutation correction, *, q < 0.05, **, q < 0.01, ***, q < 0.001.

Our previous study found that RSV infection activates the HBP pathway producing UDP-GlcNAc [17], which is a substrate and mediator of protein N-glycosylation. Therefore, we investigated the effect of KIRA8 on RSV-induced protein N-glycosylation using a lectin-enrichment/mass spectrometry approach. We identified and quantified 255 N-glycosylation sites with N-X-S/T motif (FDR 5%) (Supplemental Table S4). Among them, 167 sites were induced by RSV (Student’s *t*-test, permutation-based FDR 5%) (Figure 3A). According to cell compartment annotation, 116 out of 167 sites belong to the proteins related to ECM organization, secretion, or proteins integral to plasma membranes, such as integrins (ITGB1, ITGA5, ITGA6), laminins (LAMA3), collagens (COLA121), and ECM modifying enzymes including Procollagen-Lysine,2-Oxoglutarate 5-Dioxygenase 2 (PLOD2), Prolyl 4-Hydroxylase (P4HA1), Peroxidasin (PXDN), and proteases (cathepsin C(CTSC), TIMP metalloproteinase inhibitor (TIMP1)). Figure 3B,C show some N-glycosylated peptides that were strongly induced by RSV infection. For example, RSV induced about an 84, 12-, 16-, 15-, and 5.7-fold increase in N-glycosylation of ITG-A5 N773, Laminin Subunit Alpha 3 (LAMA3)-N600, TIMP1-N53, Thrombospondin (THBS)1-N360, and PLOD2-N209, respectively. The Panther Reactome pathway analysis of upregulated N-glycosylated proteins identified 21 enriched pathways (FDR < 0.05) (Figure 3D, Supplemental Table S5). Many of these pathways (10 out 21) are related to ECM organization and ECM–cell interaction, such as fibronectin matrix formation, laminin interactions, type I hemidesmosome assembly, syndecan interactions, ECM proteoglycans, and collagen biosynthesis and modifying enzymes. Integrins, laminins, collagens, and ECM-modifying enzymes such as PLODs, P4HA1, PXDN, and proteases are the main components of these pathways.

N-glycosylation plays an essential role in protein quality control in the ER–Golgi pathway. We found that RSV infection also altered N-glycosylation of the proteins regulating the calnexin/calreticulin cycle and ER-to-Golgi anterograde transport. For instance, N-glycosylation of glucosidase 2 subunit beta (PRKCSH), ER degradation-enhancing alpha-mannosidase-like protein 3 (EDEM3), protein sel-1 homolog 1 (SEL1L), and vesicle coating proteins such as transmembrane emp24 domain-containing protein 7 and 9 (TMED7/9) were significantly elevated in response to RSV infection. In addition, it is well-established that RSV infection induces the innate immune response. Many proteins regulating innate immunity are N-glycosylated proteins, and we found that RSV infection induced N-glycosylation on proteins involved in interleukin-4 and interleukin-13 signaling and neutrophil degranulation, such as CD44, CD59, and ICAM1.

Next, we analyzed 56 RSV-induced N-glycosylation sites that were inhibited by KIRA8. Panther Reactome pathway analysis identified 14 significantly enriched pathways, most of which involved ECM organization and integrin signaling (Figure 3E, Supplemental Table S6). We noted that FN1 matrix formation is the most significant pathway, including N glycosylated peptides ITGA5-N773 and ITGB1-N212, -N520, and -N669. As shown in Figure 3B, N-glycosylation on these sites was significantly induced by RSV infection, but KIRA8 attenuated their abundance. In addition, KIRA8 significantly reduced the N-glycosylation of proteins involved in neutrophil degranulation, such as CTSC-N53, CREG1-N160, ITGAV-N658, LAMP2-N257, GNS-N385, ASAH1-N253 and LAMP1-N103 (Figure 3F). Together, the results suggest that RSV induced aberrant N-glycosylation on ECM-related proteins and proteins regulating innate immunity is mediated by IRE1α–XBP1.

### 2.4. IRE1α–XBP1 Arm of UPR Regulates RSV-Induced Secretome

**Figure 4:**
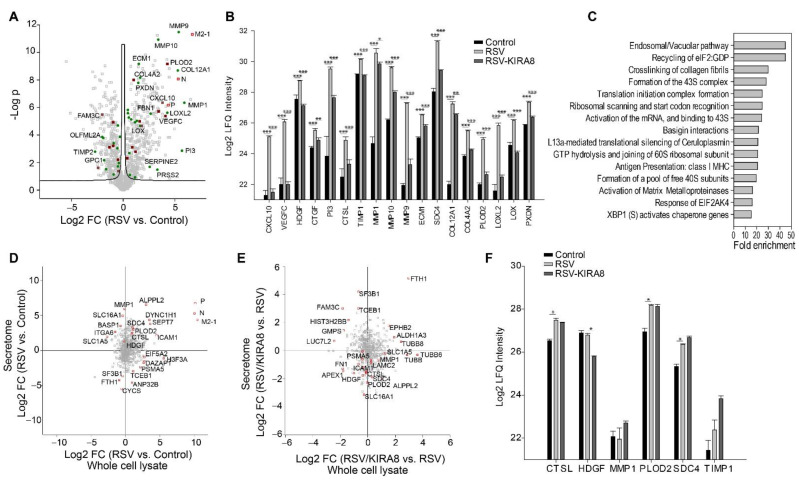
Proteomics analysis of secretome in hSAECs infected with RSV in the presence of KIRA8. hSAECs were infected with RSV at MOI 1.0 for 24 h in the presence or absence of KIRA8 (10 µM). The secretome was analyzed with label-free LC-MS/MS. (**A**) Volcano plot of secretome (RSV vs. Control). Dark red square, cytokines and growth factors; green circle, extracellular matrix proteins; and red open square, RSV proteins. (**B**) Examples of proteins whose secretion were significantly induced by RSV and blocked by KIRA8, including cytokines, growth factors, proteases, protease inhibitors, ECM, and ECM-modifying enzymes. (**C**) Pathways are strongly induced by RSV infection and regulated by the IRE1α–XBP1 arm of UPR (FDR < 0.05). (**D**) Correlation between proteome and secretome profiles (RSV vs. Control). (**E**) Correlation between proteome and secretome profiles (RSV-KIRA8 vs. RSV). (**F**) Protein expression of some proteins that were shown in Figure 4B. Student’s *t*-test with Permutation correction, *, q < 0.05, **, q < 0.01, ***, q < 0.001.

To extend our previous study of XBP1 on ECM protein secretion in mice infected with murine respiratory virus (Sendai virus), we conducted a secretome study on hSAECs infected with RSV in the presence or absence of KIRA8. A total of 1588 proteins were identified (FDR 1%). Among them, 1040 proteins were quantified (Supplemental Table S7). In response to RSV infection, the secretion of 748 proteins was significantly changed (Student’s *t*-test, permutation-based FDR *<* 5%). The secretion of RSV viral proteins, cytokines (CXCL10), growth factors (HDGF), ECM proteins (ECM1 and COL4A2), and ECM-modifying enzymes such as matrix metallopeptidases (MMP1, MMP10, and MMP9) was the most significantly induced by RSV infection (Figure 4A). For instance, the secretion of CXCL10, MMP1, MMP9, and COL12A1 was increased by 14-, 59-, 39-, and 38-fold, respectively (Figure 4A).

We identified 202 proteins whose secretion was significantly induced by RSV, but the induction was blocked by KIRA8 (Student’s *t*-test with Permutation FDR < 0.05 in both pairwise comparisons). Cytokine and growth factors (CXCL10, VEGFC, CTGF), proteases (PI3, CTSL), ECM-modifying enzymes (TIMP1, MMP1/9/10, LOXL2, PLOD2, and LOX), and collagens (COL4A2 and COL12A1) are among the proteins whose secretion were most sensitive to blockade of the IRE1α pathway (Figure 4B). We conducted a Panther Reactome pathway enrichment analysis on the top 125 proteins whose secretion was induced by RSV at least 2-fold and reduced by KIRA8 at least 2-fold. This analysis identified 31 significant pathways (Figure 4C, Supplemental Table S8). Figure 4C shows the top 15 enriched pathways. Notably, the crosslinking of collagen fibrils is one of the most enriched pathways. Our data indicate that RSV infection induced the secretion of collagen crosslinking enzymes, such as protein-lysine 6-oxidase (LOX), lysyl oxidase homolog 2 (LOXL2), lysyl hydroxylase 2 (PLOD2), and peroxidasin homolog (PXDN) (Figure 4B). Furthermore, the secretion of LOX, LOXL2, PLOD2, and PXDN was attenuated by KIRA8, suggesting that the RSV-induced secretion of these enzymes is IRE1α–XBP1 dependent. Because pathologic collagen crosslinking causes the remodeling of the airway extracellular matrix, our data suggest that the IRE1α–XBP1 arm UPR plays an important role in RSV-induced airway remodeling by regulating the secretion of collagen crosslinking enzymes, and targeting the IRE1α–XBP1 pathway may attenuate airway remodeling in RSV infection.

We also examined if the changes in the secretome were regulated by protein expression. We compared the proteome and secretome data and found that 550 proteins were quantified in the secretome study and the whole cell lysate proteome analysis. Although some proteins, such as RSV N, P, and M2-1 proteins, SEPT7, and S100A6, show a significant correlation between the changes in protein expression and secretion, most proteins exhibit a poor correlation between their secretion and expression (Figure 4D,E). The Pearson correlation of the log2 fold changes (RSV vs. control) of 550 proteins in WCL and culture medium is 0.25, and the Pearson correlation of the log2 fold changes (RSV-KIRA8 vs. RSV) of 550 proteins in WCL and culture medium is −0.04, indicating that the changes in abundance of these proteins in the culture medium are primarily regulated by secretory pathways, not by protein expression. Some of the secreted proteins shown in Figure 4B were also identified in the proteomics analysis of WCL. As shown in Figure 4F, their abundance changes in the culture medium in response to RSV infection were much greater than the changes in protein expression. For example, RSV infection did not change MMP1 protein expression but induced a 59-fold increase in secreted MMP1. Similarly, RSV infection only induced slight changes in the protein expression of CTSL, HDGF, PLOD2, and SDC4. However, the changes in their abundance in the conditioned media were much more remarkable. Together, the results suggest that targeting the secretory pathway may be a promising therapeutic strategy for virus-induced airway inflammation and remodeling.

### 2.5. IRE1α–XBP1 Arm of UPR Regulates N-Glycoprotein Secretion In Vivo

**Figure 5:**
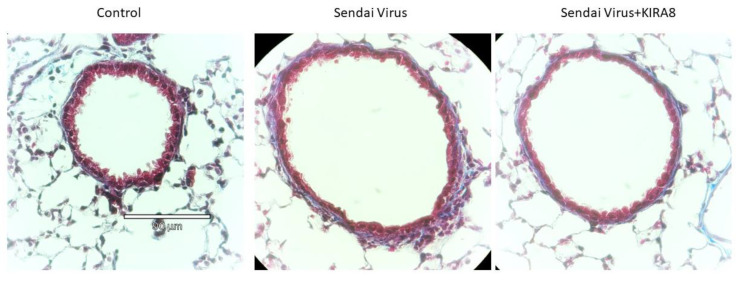
Histological analysis of IRE1α signaling in SeV infection. Masson’s trichrome staining was performed on paraffin-embedded sections from uninfected, SeV infected, or SeV+KIRA8 treated animals. Shown is a small airway. Images were taken at 40×; scalebar indicates 90 µm. Note the subepithelial accumulation of cells (nuclei) and expansion of ECM (blue) in the SeV infected mice that was reduced by KIRA8.

Sendai virus (SeV) is a negative sense, single-stranded RNA virus of the family Paramyxoviridae. SeV infection that partially mimics the pathogenesis of RSV-induced respiratory tract infections observed in humans. As with RSV, SeV replication causes inflammation, giant cell formation, and necrosis of the respiratory epithelium [22]. Our previous study shows that SeV infection in mice induces the IRE1α–XBP1 arm of the UPR in the airway, which mediates inflammatory response, HBP, and the release of ECM proteins in the mucosa in vivo. Here, we investigated how the IRE1α–XBP1 pathway regulated protein secretion in the airways of mice infected with SeV in the presence or absence of KIRA8. The bronchoalveolar lavage fluid (BALF) was collected seven days post-infection. In addition, paraffin-embedded lung tissues were sectioned and stained by Masson’s trichrome to examine changes in cellular inflammation and ECM. Here, we observed that SeV induced a subepithelial expansion of matrix and cells that was blocked by KIRA8 (Figure 5).

**Figure 6:**
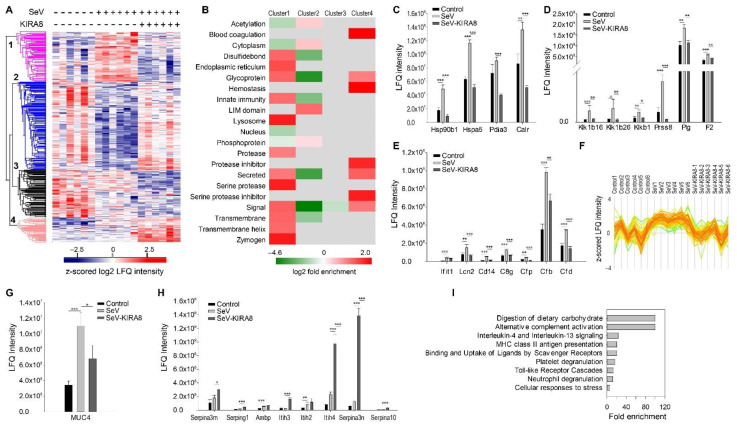
Proteomics analysis of BALF of mice infected with Sandi virus (SeV) in the presence or absence of KIRA8. The mice were infected with SeV in the presence or absence of KIRA8 (*n* = 6 in each group). The BALF was analyzed with label-free LC-MS/MS. (**A**) Unsupervised hierarchical cluster analysis of significant proteins (ANOVA with permutation-based FDR < 0.01). The colors of the heatmap represent the z-scored normalized log2 LFQ intensity of each protein. (**B**) GO annotation enrichment analysis of protein in each cluster (FDR < 0.02). The colors of heatmap represent log2 enrichment factors. Red, enrichment; green, depletion; and gray, not significant. (**C**) ER proteins in Cluster 1, whose secretion was induced by SeV and blocked by KIRA8. (**D**) Proteins related to innate immunity. (**E**) Serine proteases and peptidases. (**F**) Profiles of BALF glycoproteins in Cluster 1. (**G**) Profile of mucin-4 in BALF. (**H**) Profiles of protease inhibitors in Cluster 4, whose secretion was upregulated by KIRA8. (**I**) Pathways are strongly induced by RSV infection and regulated by the IRE1α–XBP1 arm of UPR (FDR < 0.05). Student’s *t*-test with Permutation correction, *, q < 0.05, **, q < 0.01, ***, q < 0.001.

The label-free LC-MS analysis of BALF identified 1050 proteins. Among them, 708 were quantified. Multiple sample ANOVA identified 454 significant proteins (permutation-based FDR < 0.01) (Supplemental Table S9). Unsupervised hierarchical cluster analysis of significant proteins identified four clusters (Figure 6A). We conducted an annotation enrichment analysis for proteins in each cluster. The results are shown in Figure 6B, where red indicates enrichment, green indicates depletion, and gray means that the annotation enrichment is not significant (Benjamini–Hochberg FDR < 0.02 as the cutoff for significance). In Cluster 1, where the proteins (108 proteins) were induced by SeV but blocked by KIRA8, we found that ER proteins, glycoproteins, proteins involved in innate immunity, secreted proteins (72 out of 108), and serine proteases are enriched. As shown in Figure 6C, ER proteins CLU, CALR, HSP90B1, and PIDA3 were induced by SeV and restored to the untreated level by KIRA8. In addition, we found that KIRA8 also regulated the secretion of proteins related to innate immunity. As shown in Figure 6D, SeV increased the abundance of interferon-induced protein ILIT1, neutrophil gelatinase-associated lipocalin (LCN2), monocyte differentiation antigen CD14, and complement factors (C8G, CFP, CFB, and CFD) in the alveolar space and KIRA8 reduced their secretion. Serine proteases and peptidases such as kallikrein family proteins Klk1b26, Klk1b16, KLK1B, prostasin (PRSS8), plasminogen (PLG), prothrombin (F2), and complemental factors with protease activity such as CFI, CFB, and CFD were induced by SeV, and this induction was blocked by KIRA8 (Figure 6E).

Many proteins in Cluster 1 are classic ECM factors, such as FN1, SPP1, LGALS3BP, and SFTPD (Figure 6F). In addition, we found that the level of mucin-4 was elevated in the BALF of mice infected with SeV (Figure 6G). Mucin-4 is a highly glycosylated protein that constitutes the major component of mucus. The data suggest that SeV infection induced the secretion of mucins, and the induction can be reversed by KIRA8.

The proteomics analysis of BALF confirmed that SeV increased the release of glycoproteins in the BALF, and KIRA8 restored the level of these glycoproteins to the uninfected level (Figure 6F). The unsupervised hierarchal cluster analysis indicated that glycoproteins were enriched in Cluster 1 (log2 enrichment factor 1.3, Benj. Hoch FDR 1.7 × 10^−19^); 66 out of 108 proteins in Cluster 1 are glycoproteins. On the contrary, glycoproteins were depleted in Cluster 2 (log2 enrichment factor −3.9, Benj. Hoch FDR 6.7 × 10^−24^) (Figure 6A,B), where the protein secretion was inhibited by SeV but restored by KIRA8.

Enrichment analysis of proteins in Cluster 3 yielded no significant pathway associations. Serine proteases inhibitors and proteins involved in blood coagulation were the most enriched in Cluster 4. As shown in Figure 6H, the secretion of protease inhibitors, such as alpha-1-antichymotrypsin (SERPINA3), plasma protease C1 inhibitor (SEERPING1), inter-alpha-trypsin inhibitor heavy chain H (ITIH1, ITIH2, ITIH3, and ITIH4), serine protease inhibitor A3N (SERPINA3N), and protein Z-dependent protease inhibitor (SERPINA10) were significantly increased in the mice treated with SeV and KIRA8.

Next, we conducted a Panther Reactome pathway enrichment analysis of BALF proteins whose secretion was induced by SeV at least 2-fold and reduced by KIRA8 at least 2-fold. This analysis identified nine significant pathways (Figure 6I). Notably, pathways related to innate immunity include IL4/IL13 signaling, toll-like receptor cascades, neutrophil degranulation, and alternative complement activation. This result indicates that inhibiting the IRE1α–XBP1 arm of UPR attenuated SeV-induced innate response by blocking the secretion of the mediators of these pathways.

## 3. Discussion

RSV is a ubiquitous paramyxovirus pathogen that causes childhood LRTIs and significant acute morbidity in young children [23,24]. Moreover, it is the leading cause of infant viral death worldwide [3]. In addition to their acute morbidity, severe LRTIs are associated with reshaping the pulmonary immune response, producing Th2 polarization, enhancing susceptibility to recurrent virus-induced wheezing, producing airway remodeling, and reducing lung function [25]. Our studies provide an important mechanistic understanding of why severe LRTIs are associated with the expression of ECM remodeling proteins, MMPs, and hyaluronans [12,13,14,15,16].

### 3.1. RSV-Induced Remodeling of the Basal Lamina in Chronic Airway Disease

In fatal cases of LRTI, RSV replicates in the small bronchiolar epithelium [8]. The functional role of small airway epithelial cells in RSV-induced immune response, and airway remodeling has been provided by tissue-selective genetic knockout of innate signaling in the secretoglobin (Scgb1a1) lineage of SAECs in the small airways. Here, mice deficient in NFκB signaling in Scgb1a1-derived epithelium show reduced neutrophilia, airway obstruction, and disease manifestations [26]. Moreover, systems-level findings have shown that human SAECs derived from bronchiolar epithelium produce Th2-polarizing, mucogenic, and profibrotic cytokines that mediate the pathogenesis of LRTI [27]. Recently, we found that this lineage of SAECs activates the IRE1α–XBP1 arm of UPR in response to RSV infection, which is a pathway that controls the gene expression of HBP rate-limiting enzymes and EMT core transcription regulators [16,17]. At the mechanistic level, activated XBP1s binds and recruits RNA polymerase II to the regulatory elements of IL6, SNAI1, GFPT2, and MMP9 genes. These data support the new mechanism that RSV-induced XBP1-UPR reprograms glucose metabolism, sustains the EMT process, and triggers ECM remodeling of the basal lamina.

The airway ECM is a regionally differentiated network that plays a critical role in maintaining the epithelial–mesenchymal trophic unit (EMTU) and airway physiology. In vivo, the basal lamina on which the epithelia attach is produced by combination of epithelial and subepithelial fibroblast secretion. Changes in composition, structural stiffness, and abundance of matrix-associated factors produced during injury/repair affect both components of the EMTU. Within minutes of injury, cells within the EMTU undergo induced de-differentiation and acquire enhanced motility and stem cell-like characteristics to regenerate. This complex, coordinated cellular response is mediated by matrix interactions and remodeling. Previously, we found that the RSV activation of epithelial MMP9 secretion triggered the transition of quiescent subepithelial fibroblasts into profibrotic myofibroblasts [15]. However, the global effect of RSV on ECM remodeling on cellular phenotype is not fully understood; our study extends this knowledge significantly.

Changes in the basal lamina precede other pathogenomic features of pulmonary remodeling, including smooth muscle hyperplasia, fibrosis, and inflammatory cell accumulation [28], and they correlate with the severity of disease and hyperreactivity [29]. These data indicate that remodeling the basement membrane may play an important early role in pulmonary remodeling and asthma in viral infections. The findings in this study provide a global insight into changes in ECM composition triggered by RSV-induced UPR controlling hexosamine biosynthesis and N protein glycation. Our finding that RSV induces changes in ECM composition via the IRE1α–XBP1 pathway in vitro and in vivo is a key mechanistic finding of this paper.

### 3.2. IRE1α–XBP1 Arm of the UPR Regulates Antiviral Response

Our hSAEC cellular proteomics analysis confirms that RSV infection induces the UPR, including the key ER luminal regulator HSP5A/Bip, controlling the first step in IRE1α activation for XBP1s splicing. In addition, we found that the IRE1α–XBP1 arm of the UPR plays a role in regulating the expression of nuclear pore complex (NUP35, NUP88, TPR) and mRNA export factor involved in nucleocytoplasmic transport (RAE1). The host nucleocytoplasmic trafficking system is hijacked and crucial in viral lifecycle and assembly. For instance, the RSV matrix protein (M) is localized to the nucleus early in infection, being exported to the cytoplasm later to play its central role in RSV assembly, and the disruption of nuclear export of M protein inhibits RSV assembly and reduces viral titer [30,31]. Furthermore, it has been shown that viruses target the nuclear export of mRNAs pathways to suppress antiviral response [32,33,34]. For instance, the vesicular stomatitis virus matrix (M) protein inhibits host cell gene expression by blocking bulk mRNA nuclear export [35]. The RSV nonstructural protein NS1 inhibits cellular antiviral gene expression by targeting mRNA export machinery. Previous work has shown that NS1 directly interacts with the mRNA export receptor heterodimer NXF1-NXT1 and prevents mRNA translocation through the nuclear pore complex to the cytoplasm for translation [32,34]. In this study, we found that RSV altered the expression of nuclear pore complex protein NUP35, NUP88, TPR, and mRNA export factor RAE1 in an IRE1α-dependent manner. This phenomenon may provide novel insights into how RSV regulates mRNA processing, as noted earlier in our single molecule RNA sequencing analysis [36]. The contributions of these proteins to RSV viral replication and mRNA processing will require further investigation.

In addition, our study suggests that the IRE1α–XBP1 arm of the UPR may play a role in regulating type I IFN production. IRF3, a transcription factor belonging to the IRF family, plays an essential role in antiviral response [37,38] and is rapidly induced to undergo cytoplasmic-to-nuclear translocation by RSV replication in hSAECs [39]. We found that the expression of several IRF3-mediated type I IFN genes, such as IFI6, XRCC5/Ku86, and XRCC6/Ku70, were regulated by the IRE1α/XBP1 pathway of the UPR. Ku70 and Ku86 are components of the DNA-dependent protein kinase complex, which is a DNA sensor for activating IRF-3-dependent innate immunity [40]. In addition, viral infection induces the interaction of Ku70 with the adaptor proteins STING, which is a well-characterized mediator of type I IFN production [41].

### 3.3. IRE1α–XBP1 Arm of the UPR Regulates N-Glycosylation in Response to RSV Infection

The HBP is a homeostatic response to TGFβ or viral infection, increasing the cellular capacity for N-glycosylation and improving protein quality control [17,42]. Mechanistically, we provide evidence that RSV perturbs glycolysis via the HBP in hSAECs, enhancing UDP-GlcNAc accumulation and protein N-glycosylation in an IRE1α-dependent manner. N-glycosylation is important for cellular proteostasis and virion assembly by promoting the processing of RSV F and G glycoproteins [43].

**Figure 7:**
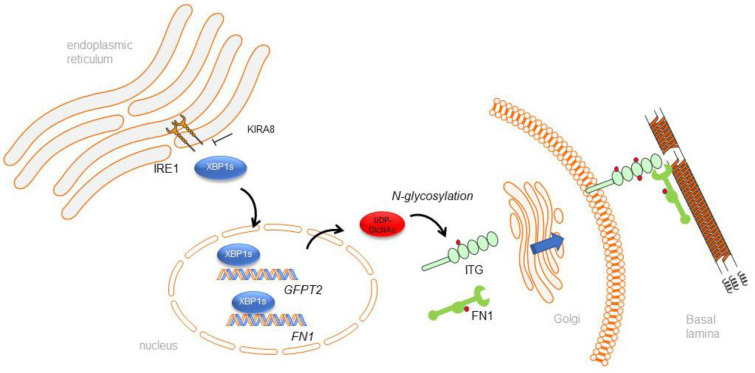
RSV induced N-glycosylation is mediated by the IRE1α–XBP1 arm of the UPR. A schematic view of the relationship between the IRE1α–XBP1 pathway of the unfolded protein response, accumulation of UDP-GlcNAc, protein N glycosylation, and remodeling of the basal lamina. IRE1 activated in the ER induces alternative splicing and produces the formation of activated XBP1s, which is a transcription factor controlling the expression of the hexosamine biosynthetic pathway, integrin (ITG), and ECM components, including fibronectin 1 (FN1). UDP-GlcNAc is a rate-limiting enzyme for protein N-glycosylation. After processing through the Golgi, glycosylated ECM components are presented on the cell surface and contribute to remodeling of the basal lamina.

This glycoproteomics analysis shows that RSV infection increases N-glycosylation of the integrins (ITGB1, ITGA5, ITGA6), laminins (LAMA3), collagens (COLA121), and ECM-modifying enzymes such as PLODs, P4HA1, PXDN, and proteases (CTSC, TIMP1) in an IRE1α-dependent manner (schematically illustrated in Figure 7). These proteins are essential for ECM organization, ECM–cell signaling, and neutrophil degranulation. N-glycosylation is not only critical for protein folding and quality control but also an important post-translational modification for signaling transduction. For instance, integrins constitute a significant family of cell-surface-adhesion receptors, linking cells to ECM and other surrounding cells [44]. In addition to performing a structural role, integrins function as signal transducers, participating in various intracellular signaling pathways [44,45,46]. Integrin N-glycosylation has been shown to be critical for function, where aberrant integrin N-glycosylation alters growth factor signaling pathways associated with fatal interstitial lung disease and metastatic cancers [45,46,47,48,49,50].

### 3.4. IRE1α–XBP1 Arm of the UPR Regulates RSV Secretome

We previously reported that the IRE1α–XBP1 arm of UPR regulates ECM secretion in airway epithelial cells undergoing EMT [17,42]. This study found that the IRE1α–XBP1 arm of UPR also plays a significant role in regulating secretory pathways in airway epithelial cells infected with RSV. The secretion of cytokine and growth factors (CXCL10, VEGFC, CTGF), proteases (PI3, CTSL), ECM-modifying enzymes (TIMP1, MMP1/9/10, LOXL2, PLOD2, and LOX), and collagens (COL4A2 and COL12A1) is IRE1α-dependent, and their secretion can be blocked by IRE1α inhibitor, KIRA8. 

Our data indicate that crosslinking collagen fibrils is one of the most significant pathways mediated by the IRE1α–XBP1 arm of the UPR. The secretion of collagen crosslinking enzymes, such as LOX, LOXL2, PLOD2, and PXDN, was markedly induced by RSV infection, and KIRA8 blocked this induction. More importantly, the secretion of these enzymes was primarily regulated by the secretory pathways, independent of protein expression. LOX and LOXL2 are lysyl oxidases, which are essential for the normal development and function of the respiratory system and the integrity of elastic and collagen fibers in various tissues [51,52]. When secreted into the extracellular matrix, LOX and LOXL2 promote the crosslinking of ECM by mediating oxidative deamination of peptidyl lysine residues in precursors to fibrous collagen and elastin [52]. PLOD2 is lysyl hydroxylase, forming hydroxylysine residues in -Xaa-Lys-Gly- sequences in collagens. These hydroxylysines serve as attachment sites for carbohydrate units and are essential for the stability of the intermolecular collagen crosslinks [53]. Aberrant lysyl hydroxylation and collagen crosslinking contribute to the progression of many collagen-related diseases, such as fibrosis and cancer [54]. PXDN can also stabilize the ECM by protein crosslinking and plays an important role in fibrosis [55,56]. Pathologic collagen crosslinking causes the remodeling of the airway extracellular matrix, and our data indicated that the secretion of these enzymes could be attenuated by inhibiting the IRE1α–XBP1 arm of UPR, suggesting that targeting the IRE1α–XBP1 arm of UPR has a potential therapeutical value for treating or preventing RSV-induced airway remodeling.

### 3.5. IRE1α–XBP1 Arm of UPR Regulates ECM and Mediators of Innate Immunity In Vivo

Consistent with our in vitro studies, we found that the IRE1α–XBP1 arm of UPR regulates ECM secretion in the BALF of the SeV-infected mouse. In addition, the IRE1α–XBP1 arm of UPR also played a role in regulating mediators of complement pathways, IL4/IL13 pathway, and neutrophil degranulation. In our previous study, we found that HBP activation in the lung of mice infected with SeV and inhibiting IRE1a blocked it. In this study, we found that SeV induced the secretion of glycoproteins to BALF, and KIRA8 attenuated their secretion, confirming that the IRE1α–XBP1 arm of UPR regulated the activation of HBP in vivo and glycoprotein metabolism. 

We found that the secretion of serine proteases and peptidases in BALF was significantly induced by SeV infection and attenuated by KIRA8. Furthermore, KIRA8 strongly induced the secretion of serine protease inhibitors. Proteases and protease inhibitors in the normal lungs coordinate their functions in lung injury and repair [57,58]. Dysregulation of the proteases–antiproteases balance is crucial in the manifestation of different types of lung diseases, such as chronic obstructive pulmonary disease (COPD), asthma, cystic fibrosis, and acute respiratory distress syndrome, where a marked increase in protease activities was observed [59,60,61,62,63]. Inhibiting protease activity has been explored for treating airway inflammation and remodeling diseases [63,64]. Our study provided preliminary evidence that inhibiting IRE1α can attenuate the secretion of proteases while inducing the secretion of proteases inhibitors. It will require further investigation to determine whether targeting the IRE1α–XBP1 arm of UPR can restore the proteases–antiproteases balance in the lung and reduce airway inflammation and remodeling.

## 4. Materials and Methods

### 4.1. Human Small Airway Epithelial Cell (hSAEC) Culture and Treatment

hSAECs are immortalized primary human small airway epithelial cells [65] from ATCC (PCS-301-010, at passage 2). hSAECs were grown in SAGM small airway epithelial cell growth medium (Lonza, Walkersville, MD, USA) and used at passage 4. hSAECs undergo growth factor-induced cell-state transition [66] and maintain RSV-induced genomic and proteomic signatures representative of primary cells [27]. The human RSV long strain was grown in Hep-2 cells, prepared by sucrose cushion purification, tittered by methylcellulose plaque assay [26,67] and quick-frozen until use. The selective IRE1α RNAse inhibitor KIRA8 (MedChemExpress, South Brunswick Township, NJ, USA) [68] was applied to the cells 2 h prior to RSV infection.

### 4.2. Immunofluorescence of ECM Deposition

Glass coverslips were sequentially coated in 0.1 mg/mL PDL and 0.1% gelatin and washed in PBS three times after each coating. 3 × 10^5^ SAECs per well were seeded onto the coverslips in a 24-well plate. The cells were cultured for three days to reach high confluency. The cells were pre-treated for two h in DMSO or 10 µM KIRA8, which was followed by mock or RSV infection (1.0 MOI) for 24 h. After washing in PBS, the cells were either fixed in 4% paraformaldehyde (10 min at RT) or subjected to decellularization. For decellularization, the cells were incubated in 0.2% Triton X-100 in PBS for 15 min at RT, which was followed by brief incubation in 20 mM NH_4_OH in PBS. After washing in PBS, the ECM was fixed in 4% paraformaldehyde (10 min at RT). Both fixed cells and ECM were blocked in 10% goat serum (GS) in PBS for 1 h and then incubated with anti-FN antibody (ab2413, 1:400) in the blocking buffer overnight at 4 °C. After washing in PBS three times (5 min each time), Alexa Fluor 647-goat anti-rabbit IgG (Thermo, Waltham, MA, USA, 1:1000) was applied and incubated for one hour at RT. After washing in PBS three times (5 min each time), the coverslips were mounted with ProLong Gold Antifade Mountant with DAPI (Thermo, Waltham, MA, USA).

### 4.3. Protein Extraction and Trypsin Digestion

Washed hSAECs were extracted with Trizol reagent (Invitrogen, Carlsbad, CA, USA) [42,69]. The protein pellet was resuspended in 100 μL of 8 M Guanidine HCl. The protein concentration was measured using BCA assay. One milligram of proteins from each sample was processed for digestion. The proteins were first reduced with 10 mM DTT at room temperature for 30 min, which was followed by alkylation with 30 mM iodoacetamide at room temperature for two hours. The sample was then diluted with 200 µL of 50 mM ammonium bicarbonate (pH 8.0). An aliquot of Lys-C/Trypsin solution (Promega, Madison, WI, USA) was added to each sample at the 50:1 protein:enzyme ratio. The samples were incubated at 37 °C overnight, and the solutions were further diluted with 500 µL of 100 µM of triethylammonium. An aliquot of Trypsin solution (Promega, Madison, WI, USA) was added to each sample at the 50:1 protein:enzyme ratio. The samples were incubated at 37 °C for 16 h. The trypsin digestion was stopped by adding 100 µL of 10% trifluoroacetic acid to each sample. Ten micrograms of tryptic peptides were desalted on reversed-phase tC18 SepPak columns (Waters, Milford, MA, USA) and analyzed by LC-MS/MS. 

### 4.4. Enrichment of N-Glycosylation

N-glycosylation enrichment was performed according to the N-glyco-FASP protocol of Wisniewski et al. [70]. Briefly, 100 μg peptides were vacuum dried and dissolved in the binding buffer (1 mM CaCl_2_, 1 mM MnCl_2_, 0.5 M NaCl in 20 mM Tris-HCl, pH = 7.3). Samples were heated for 10 min at 95 °C and cooled down to room temperature. Lectin mixture (90 μg ConA, 90 μg WGA, 71.5 μg RCA120) was added to the sample and incubate at room temperature for 1 h. Samples were transferred to YM-30 filter units (Microcon, Millipore, Burlington, MA, USA), and the unbound peptides were eluted by centrifugation at 10,000× *g* for 10 min. The captured peptides were washed four times with 200 μL of binding buffer and twice with 50 μL of 40 mM NH_4_HCO_3_. Peptides were incubated with 2 μL N-glycosidase F (Roche, Basel, Switzerland) in 40 μL of 40 mM NH_4_HCO_3_ at 37 °C for 3 h. The released deglycosylated peptides were eluted with 40 mM NH_4_HCO_3_. The eluted peptides were dried by speedvac and acidified with 0.01% trifluoroacetic acid. Peptides were desalted on Ziptip C18 (Waters, Millford, MA, USA).

### 4.5. NanoLC-MS/MS Analysis

The desalted peptides were reconstituted in 20 μL 4% ACN/0.1% formic acid. All peptide samples were separated on an online nanoflow Easy nLC1000 UHPLC system (Thermo Scientific, Waltham, MA, USA) and analyzed on a Q Exactive Orbitrap mass spectrometer (Thermo Scientific, San Jose, CA, USA). Then, 10 µL of the sample was injected onto a capillary peptide trap column (Acclaim^®^ Pepmap 100, 75 µm × 2 cm, C18, 3 µm, 100 Å, Thermo Scientific, Waltham, MA, USA). After sample injection, the peptides were separated on a 25 cm UHPLC reversed-phase column (Acclaim^®^ Pepmap 100, 75 µm × 25 cm, C18, 2 µm, 100 Å, Thermo Scientific, Waltham, MA, USA) at a flow rate of 300 nL/min. A 4 h linear gradient from 2% solvent A (0.1% formic acid in H_2_O) to 35% solvent B (0.1% formic acid in ACN) was used for each LC-MS/MS run. The data-dependent acquisition was performed using the Xcalibur 2.3 software in positive ion mode at a spray voltage of 2.1 kV. Survey spectra were acquired in the Orbitrap with a resolution of 70,000, the maximum injection time of 20 ms, an automatic gain control (AGC) of 1e6, and a mass range from 350 to 1600 *m/z*. The top 15 ions in each survey scan were selected for higher-energy collisional dissociation scans with a resolution of 17,500. For all higher-energy collisional dissociation scans, collision energy was set to 28, the maximum inject time was 200 ms, and the AGC was 1e5. Ions selected for MS/MS were dynamically excluded for 30 s after fragmentation.

### 4.6. Proteomics Data Analysis and Statistical Analysis

The mass spectrometry data was analyzed with MaxQuant (Version 1.5.2.8) [71] as described previously [17,42]. We used the Perseus platform [72] to analyze the Maxquant output, including statistics, hierarchical clustering, and principal component analysis (PCA). Reversed identifications and proteins identified only by site modification were strictly excluded from further analysis. For proteomics analysis, proteins identified only by site modification were excluded from further analysis as well. After filtering (3 valid values in at least one group), the remaining missing values were imputed from a normal distribution (width: 0.3 of standard deviation; downshift: 1.8 of standard deviation). A multiple-sample ANOVA test with permutation-based FDR correction and a two-way ANOVA test with permutation-based FDR was performed to identify the significantly differentially expressed proteins. The unsupervised hierarchical clustering and heat map were based on protein LFQ intensity or the MS intensity of N-glycosylated peptides. The rows of the heat map indicate the proteins, and the columns indicate the samples. The log2 LFQ intensity of each protein was z-score normalized for each row and subjected to hierarchical clustering using Euclidean distances between means. Genome ontology enrichment analysis of molecular functions and biological function in differentially expressed proteins was completed using Panther (http://pantherdb.org/, accessed on 7 December 2021). This classification uses an evolutionary framework to infer protein functions in a species-independent manner [73]. The resulting *p*-values were adjusted with Bonferroni correction for multiple testing. The significant hits are those with the adjusted *p*-valve better than 0.05.

### 4.7. RNA Isolation and qRT-PCR

Total cellular RNA was isolated using RNeasy kit with on-column DNase digestion (Qiagen). The synthesis of complementary DNAs (cDNAs) was completed with a First Strand cDNA Synthesis Kit (Thermo Scientific). qRT-PCR assays were performed using a SYBR Green Master mix (Bio-Rad, Hercules, CA, USA) and gene-specific primers as described previously [17]. Data are presented as fold change using the ΔΔCt method.

### 4.8. Murine Respirovirus (Sendai Virus (SeV)) Infection

Animal experiments were performed according to the NIH Guide for Care and Use of Experimental Animals and approved by the University of Wisconsin at Madison Institutional Animal Care and Use Committee (approval no. M006067-R01). Wild-type 7-week old C57BL/J6 black mice (both genders) (*n* = 12) were administered Sendai virus (SeV, 104 PFU, Cantell Strain, ATCC) or vehicle (PBS) via the intranasal route. Randomly selected six SeV-infected mice were treated every day with KIRA8 (50 mg/kg/day; MedChemExpress, South Brunswick Township, NJ, USA) for 3 days via the intraperitoneal route starting 24 h after SeV infection. KIRA8 solution at 6 mg/mL was prepared using 10% DMSO/90% corn oil formula and maintained at 37 °C for smooth injection. The mice were euthanized on day 7 post-infection. In a separate study, wild-type 7-week old C57BL/J6 black mice (*n* = 6) were administered vehicle (PBS), used as the controls, and euthanized on day 7. Bronchoalveolar lavage fluid was collected from these mice and used for proteomic analysis. Proteins in 80 microliters of BALF were first reduced with 10 mM dithiothreitol (DTT) and alkylated with 30 mM iodoacetamide. Next, the proteins were first digested with LysC-trypsin (Promega, Madison, WI, USA), which was followed by trypsin (Promega, Madison, WI, USA). The peptides were desalted and analyzed by NanoLC-MS/MS as described above.

For histological analysis, mouse left lungs were inflated with 4% neutral buffered formalin (Fisherbrand, Pittsburg, PA, USA) and fixed for 24–48 h before being submerged in 70% ethanol, paraffin-embedded and sectioned. Serial lung sections were then subjected to either Masson’s trichrome staining. Shown are images at 40× magnification.

## 5. Conclusions

We conclude that the IRE1α–XBP1s pathway regulates RSV-induced innate immunity and the secretion of ECM proteins in cellulo and in vivo. These secreted ECM proteins are N-glycosylated and function in the organization, secretion, and modification of the ECM of the basal lamina. Our data further indicate that these proteins are largely controlled by a viral inducible secretory pathway that functions in parallel with changes in protein synthesis. These data provide novel, mechanistic insights into how paramyxovirus infections provoke airway remodeling by perturbation of the basal lamina.

## Data Availability

The mass spectrometry proteomics data have been deposited to the ProteomeXchange Consortium via the PRIDE partner repository with the dataset identifier PXD034780 (https://www.ebi.ac.uk/pride/, accessed on 21 June 2022).

## Supplementary Materials

The supporting information can be downloaded at: https://www.mdpi.com/article/10.3390/ijms23169000/s1.
